# Association of serum selenium with anemia‐related indicators and risk of anemia

**DOI:** 10.1002/fsn3.2261

**Published:** 2021-03-27

**Authors:** Qing Zhou, Baozhu Zhang, Xi Chen, Qiuyan Chen, Lu Hao

**Affiliations:** ^1^ Central Laboratory People’s Hospital of Baoan District The Second Affiliated Hospital of Shenzhen University Shenzhen China; ^2^ Department of Oncology People’s Hospital of Baoan District The Second Affiliated Hospital of Shenzhen University Shenzhen China; ^3^ Science and Education Department Shenzhen Baoan Shiyan People’s Hospital Shenzhen China

**Keywords:** anemia, cross‐sectional study, hemoglobin concentrations, Selenium

## Abstract

Few studies have examined the association of serum selenium with anemia‐related indicators and risk of anemia. We conducted a cross‐sectional analysis of 2,902 adults in 2003–2004 National Health and Nutrition Examination Survey database. Multivariable linear and logistic regression models were used to examine the association of serum selenium with anemia‐related indicators and risk of anemia. The nonlinear relationship was analyzed using a generalized additive model with the smoothing plot. A total of 1,472 males and 1,430 females with a mean age of 61.94 ± 13.73 years were included. Compared with the lowest quintile, the highest quintile of serum selenium was associated with increased level of serum iron (β = 12.44, 95% confidence interval [CI]: 7.14, 17.75, *p* < .001), mean corpuscular hemoglobin concentration (MCHC) (β = 0.14, 95%CI: 0.02, 0.26, *p* = .020), and hemoglobin (β = 0.40, 95%CI: 0.19, 0.61, *p* < .001), and decreased risk of anemia (odds ratio [OR] = 0.47, 95%CI: 0.28, 0.77, *p* = .002). Furthermore, smoothed plots suggested the nonlinear relationships between serum selenium and MCHC, hemoglobin level, and risk of anemia. Interestingly, on the left of inflection point, serum selenium was associated with decreased risk of anemia (OR = 0.972, 95%CI: 0.960, 0.985, *p* < .001), and then, the risk of anemia increased with increasing serum selenium concentration (OR = 1.011, 95%CI: 1.002, 1.021, *p* = .023). Future large‐scale, polycentric prospective studies should be conducted to verify our results.

## Graphical abstract

Higher serum selenium is associated with an increased serum iron level, MCHC, and hemoglobin level and a decreased risk of anemia. Due to the narrow physiological range of selenium, the relationships between selenium‐ and anemia‐related indicators and risk of anemia were nonlinear. Our findings would provide new insights on selenium nutrition that implicate anemia and human health.

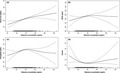

## 1 INTRODUCTION

Anemia continues to be a crucial public health problem causing 68.36 million years lived with disability (Kassebaum et al., 2014). The prevention and control of anemia is one of the WHO 2025 global nutrition targets (Garcia‐Casal et al., 2019). Anemia is a condition in which the number or oxygen‐carrying capacity of red blood cells is insufficient to meet normal physiological needs (Cusick et al., 2008). Iron deficiency is considered to be the most common cause of anemia worldwide, but the lack of other vitamins or the presence of inflammation, infection, or inherited hemoglobin disorders can also lead to anemia (Cheng et al., 2012; Means, 2000; Weiss et al., 2019; World Health Organization (WHO), 2001). A previous study demonstrated that anemia is not only a multifactorial disease but also a risk factor that affects the occurrence of diseases involving the nervous system, respiratory and circulatory system, skin and mucous membrane, digestive system, and endocrine system (Y. Hu, Li, et al., 2019). These reasons explain why anemia has been an ongoing public health and clinical problem that has attracted considerable attention.

Selenium is an essential trace element for human functioning, and the lack of selenium often reduces glutathione peroxidase activity (Kryscio et al., 2017). Many studies have showed that selenium plays an important role in antioxidation, immunity, and anti‐inflammation through the activities of selenium‐dependent glutathione peroxidase (GPX) and other selenoproteins (Klein et al., 2011; Kryscio et al., 2017; Lin & Shen, 2021; Razavi et al., 2016; Tamtaji et al., 2019). Studies have shown that adequate selenium can ensure one's health with respect to various hematological indicators, including red blood cell count and hemoglobin concentration (Gibson et al., 2011). Hemoglobin that carries Fe^3+^ is called methemoglobin, which cannot carry oxygen. Glutathione peroxidase can prevent the production of methemoglobin (Sazawal et al., 2010). Few studies had comprehensively explored the association between the levels of serum selenium and anemia, and the evidence on this topic has remained inconsistent. Cross‐sectional studies have demonstrated that a low selenium level is independently associated with the likelihood of anemia among school children and older men and women (Nhien et al., 2008; Semba et al., 2006, 2009). However, several lines of evidence have suggested no significant association between serum selenium and hemoglobin levels (Diana et al., 2019; Wai et al., 2020).

Recent studies have revealed a nonlinear association of circulating selenium levels with the incidences of dyslipidemia and diabetes in adults in the United States (Huang et al., 2020; Lin & Shen, 2021). Furthermore, previous studies have discovered that low and high serum selenium levels (<130 and > 150 ng/ml, respectively) are negatively and positively associated with mortality, respectively (Bleys et al., 2008). These findings suggest that the health relationship of circulating selenium is complicated, and the health outcome may be affected by the differences in background selenium levels. However, no study has explored the nonlinear relationship between serum selenium level and risk of anemia.

Thus, our study investigated the relationship of serum selenium level with anemia‐related indicators and the risk of anemia through analysis of data in the 2003–2004 National Health and Nutrition Examination Survey (NHANES) database. Furthermore, we used a generalized additive model (GAM) to investigate the nonlinearity of the aforementioned relationship.

## 2 MATERIALS AND METHODS

### 2.1 Study population

We used data from the NHANES 2003–2004, which were conducted by the National Center for Health Statistics of the Centers for Disease Control and Prevention (CDC). In NHANES 2003–2004, there were a total of 10,122 individuals, and our analysis was limited 5,620 individuals aged 18 years old. Among them, the individuals without complete serum selenium data (*n* = 2,718) were further excluded. In the end, a total of 2,902 individuals were included in this cross‐sectional study. The NHANES protocols were approved by the national center for health statistics research ethics review board, and written informed consent from all the participants was provided during the survey.

### 2.2 Measurement of serum selenium and anemia‐related indicators

The serum selenium was measured in serum by inductively coupled plasma dynamic reaction cell spectrometry using methane as reaction gas. Serum iron was measured at the Collaborative Laboratory Services in Ottumwa, Iowa. The method used to measure the serum iron concentration was a timed‐endpoint method. Iron was released from transferrin by acetic acid and reduced to the ferrous state by hydroxylamine and thioglycolate. The ferrous ion was complexed with the FerroZine Iron Reagent. The system monitored the change in absorbance at 560 nm at a fixed time interval. This change in absorbance was directly proportional to the concentration of iron in the sample. The methods used to derive complete blood count parameters were based on the Beckman Coulter method of counting and sizing, in combination with an automatic diluting and mixing device for sample processing, and a single beam photometer for hemoglobinometry.

### 2.3 Outcome definition

Hemoglobin concentration was divided into normal (hemoglobin: ≥130 g/L for males and ≥ 120 g/L for females) and anemia group (hemoglobin: <130 g/L for males and < 120 g/L for females) according to the WHO diagnostic criteria of anemia (World Health Organization (WHO), 2001).

### 2.4 Statistical Analysis

The means and standard deviations (SDs) were used to describe normally distributed continuous variables. Categorical variables were expressed as numbers and percentages. Differences between means were compared by analysis of variance (ANAOV), and categorical variables were compared by chi‐square test. We applied multiple linear or logistic regression model to estimate the independent associations of serum selenium concentration with hematological indices of anemia after adjusting for age, gender, education, race, body mass index, energy intake, dietary fiber, and dietary iron. We used GAM to identify the nonlinear relationship between serum selenium and hematological indices of anemia. If the nonlinear correlation was observed, the piecewise linear regression model was used to examine the threshold effect of serum selenium on the hematological indices of anemia by the smoothing plot. Furthermore, we conducted the log likelihood ratio test comparing linear regression model with two piecewise linear regression model. All analyses were performed using R (http:// www.R‐project.org). A *p*‐value < .05 was considered statistically significant.

## 3 RESULTS

The demographic and clinical characteristics of study population are presented in Table 1. The study included 2,902 participants (1,472 males and 1,430 females) with a mean age of 61.94 ± 13.73 years. The mean hemoglobin level was 14.30 ± 1.50 g/dl. The mean serum iron level was 83.42 ± 32.81 μg/dl. The percentage of anemia was 8.51%. The subjects were divided into five groups by the quintile of serum selenium concentration. Participants with higher serum selenium concentration (Q2‐Q5) had higher hemoglobin and serum iron level and lower risk of anemia compared to those with lowest serum selenium (Q1).

**TABLE 1:** General characteristics of study population, presented by serum selenium category^a^

Serum selenium	Total	Q1 (<120 ng/ml)	Q2 (121–130 ng/ml)	Q3 (131–138 ng/ml)	Q4 (139–149 ng/ml)	Q5 (>150 ng/ml)	*p*‐value^c^
*N*	2,902	531	603	592	583	593	
Age, years ^a^	61.94 ± 13.73	61.86 ± 14.06^1^	60.98 ± 14.20	62.36 ± 13.39	61.57 ± 13.57	62.91 ± 13.40	.141
Male, %^b^	1,472 (50.71)	219 (41.17)	260 (43.12)	305 (51.52)	316 (54.20)	331 (55.82)	<.001
Race, %							
Non‐Hispanic White	1644 (56.63)	303 (56.95)^2^	311 (51.58)	326 (55.07)	345 (59.18)	359 (60.54)	<.001
Black	510 (17.57)	147 (27.63)	134 (22.22)	98 (16.55)	73 (12.52)	58 (9.78)	
Mexican American	561 (19.32)	44 (8.27)	118 (19.57)	128 (21.62)	129 (22.13)	142 (23.95)	
Other Hispanic	76 (2.62)	14 (2.63)	21 (3.48)	13 (2.20)	22 (3.77)	6 (1.01)	
Other Race/Ethnicity	112 (3.86)	24 (4.51)	19 (3.15)	27 (4.56)	14 (2.40)	28 (4.72)	
Some College or college graduate, %	1,227 (42.37)	203 (38.59)	247 (40.96)	265 (44.76)	243 (41.68)	269 (45.44)	.119
BMI, kg/m^2^	28.69 ± 5.89	29.07 ± 6.94	29.11 ± 6.00	28.74 ± 5.83	28.59 ± 5.31	27.97 ± 5.27	.007
Hemoglobin, g/dl	14.30 ± 1.50	13.90 ± 1.66	14.15 ± 1.46	14.38 ± 1.52	14.54 ± 1.36	14.51 ± 1.41	<.001
Serum iron, μg/dl	83.42 ± 32.81	76.98 ± 35.71	80.41 ± 32.77	83.75 ± 33.73	86.23 ± 30.86	89.21 ± 29.68	<.001
MCHC, g/dl	33.71 ± 0.75	33.58 ± 0.84	33.67 ± 0.78	33.70 ± 0.72	33.77 ± 0.69	33.80 ± 0.70	<.001
CRP, mg/dl	0.52 ± 1.11	0.89 ± 2.12	0.52 ± 0.81	0.44 ± 0.65	0.43 ± 0.64	0.37 ± 0.63	<.001
Energy, kcal	1907.59 ± 768.84	1826.84 ± 712.72	1812.22 ± 728.36	1967.47 ± 795.20	1972.79 ± 810.01	1951.31 ± 774.59	<.001
Dietary fiber, g	15.42 ± 8.06	13.40 ± 6.58	14.93 ± 7.37	15.56 ± 7.86	16.25 ± 8.84	16.71 ± 8.91	<.001
Dietary iron, mg	14.77 ± 7.36	13.54 ± 6.55	14.25 ± 7.18	15.00 ± 7.03	15.72 ± 8.08	15.24 ± 7.63	<.001
Anemia, %	247 (8.51)	73 (13.75)	53 (8.79)	41 (6.93)	36 (6.17)	44 (7.42)	<.001

Abbreviations: BMI, body mass index; CRP, C‐reactive protein;MCHC, mean corpuscular hemoglobin concentration; Q, quintile;TIBC, total iron‐binding capacity; TS, transferrin saturation.

^a^ Data are expressed as mean ± standard deviation (*SD*).

^b^ *n* (%).

^c^ *p* values indicate significant differences between the groups by ANOVA or chi‐square tests.

Table 2 shows the unadjusted and fully adjusted associations of serum selenium concentration with hematological indices of anemia. In multivariate‐adjusted models, the highest quintile of serum selenium was associated with increased level of serum iron (β = 12.44, 95%CI: 7.14, 17.75, *p* < .001), MCHC (β = 0.14, 95%CI: 0.02, 0.26, *p* = .020), and hemoglobin (β = 0.40, 95%CI: 0.19, 0.61, *p* < .001) compared with the lowest quintile. Furthermore, the highest quintile of serum selenium was associated with decreased risk of anemia compared with the lowest quintile (OR = 0.47, 95%CI: 0.28, 0.77, *p* = .002). Participants tended to have a higher serum iron, MCHC, and hemoglobin level and lower risk of anemia as the quintile of serum selenium concentration increased (all *p* for trend < .05).

**TABLE 2:** Multivariate regression for associations between serum selenium and hematological indices of anemia

Serum selenium, ng/ml	Model 1^a^		Model 2^b^		Model 3^c^	
	β/OR (95%CI)	*p*‐value	β/OR (95%CI)	*p*‐value	β/OR (95%CI)	*p*‐value
Serum iron, μg/dl						
Serum selenium, quintile						
Q1 (<120 ng/ml)	Reference		Reference		Reference	
Q2 (121–130 ng/ml)	4.15 (−1.43, 9.73)	.145	3.41 (−2.06, 8.88)	.222	3.82 (−1.82, 9.46)	.185
Q3 (131–138 ng/ml)	7.26 (1.93, 12.58)	.008	5.91 (0.69, 11.12)	.027	6.67 (1.30, 12.04)	.015
Q4 (139–149 ng/ml)	10.73 (5.49, 15.96)	<.001	7.77 (2.61, 12.93)	.0032	8.80 (3.49, 14.10)	.001
Q5 (>150 ng/ml)	15.04 (9.85, 20.23)	<.001	11.08 (5.93, 16.23)	<.001	12.44 (7.14, 17.75)	<.001
*p* for trend	<.001		<.001		<.001	
MCHC, g/dl						
Serum selenium, quintile						
Q1 (<120 ng/ml)	Reference		Reference		Reference	
Q2 (121–130 ng/ml)	0.08 (−0.04, 0.21) 0.2025	.203	0.04 (−0.09, 0.16)	.561	0.02 (−0.11, 0.15)	.785
Q3 (131–138 ng/ml)	0.17 (0.05, 0.29)	.006	0.11 (−0.01, 0.23)	.069	0.09 (−0.04, 0.21)	.165
Q4 (139–149 ng/ml)	0.20 (0.08, 0.32)	<.001	0.12 (0.01, 0.24)	.040	0.09 (−0.03, 0.21)	.145
Q5 (>150 ng/ml)	0.30 (0.18, 0.42)	<.001	0.18 (0.06, 0.29)	.002	0.14 (0.02, 0.26)	.020
*P* for trend	<.001		.001		.015	
Hemoglobin, g/dl						
Serum selenium, quintile						
Q1 (<120 ng/ml)	Reference		Reference		Reference	
Q2 (121–130 ng/ml)	0.36 (0.10, 0.61)	.006	0.26 (0.04, 0.47)	.021	0.23 (0.00, 0.45)	.048
Q3 (131–138 ng/ml)	0.49 (0.25, 0.73)	<.001	0.32 (0.11, 0.53)	.002	0.31 (0.10, 0.52)	.004
Q4 (139–149 ng/ml)	0.79 (0.55, 1.03)	<.001	0.46 (0.26, 0.67)	<.001	0.46 (0.25, 0.67)	<.001
Q5 (>150 ng/ml)	0.84 (0.60, 1.08)	<.001	0.40 (0.20, 0.60)	<.001	0.40 (0.19, 0.61)	<.001
*P* for trend	<.001		<.001		<.001	
Risk of anemia						
Serum selenium, quintile						
Q1 (<120 ng/ml)	Reference		Reference		Reference	
Q2 (121–130 ng/ml)	0.49 (0.31, 0.79)	.003	0.64 (0.39, 1.06)	.081	0.65 (0.39, 1.10)	.107
Q3 (131–138 ng/ml)	0.37 (0.24, 0.59)	<.001	0.45 (0.28, 0.72)	.001	0.45 (0.27, 0.75)	.002
Q4 (139–149 ng/ml)	0.24 (0.15, 0.38)	<.001	0.32 (0.19, 0.52)	<.001	0.32 (0.19, 0.54)	<.001
Q5 (>150 ng/ml)	0.32 (0.20, 0.50)	<.001	0.46 (0.28, 0.74)	.001	0.47 (0.28, 0.77)	.003
*P* for trend	<.001		0.010		0.018	

Abbreviations: OR, odds ratio; 95% CI, 95% confidence intervals; MCHC, mean corpuscular hemoglobin concentration;

^a^ Model 1: Nonadjusted;

^b^ Model 2: Adjusted for age, gender, education (less than high school, high school, more than high school, or missing), race (non‐Hispanic white, black, Mexican American, other Hispanic, other race/ethnicity or missing), and body mass index (<18.5, 18.5–24.9, 25–29.9, >30, or missing);

^c^ Model 3: Adjusted for age, gender, education (less than high school, high school, more than high school, or missing), race (non‐Hispanic white, black, Mexican American, other Hispanic, other race/ethnicity or missing), body mass index (<18.5, 18.5–24.9, 25–29.9, >30, or missing), energy intake, dietary fiber, and dietary iron.

Table 3 shows the associations between serum selenium and hematological indices of anemia in different subgroups. Serum selenium was positively associated with serum iron level in both genders and participants with high C‐reactive protein level and age less than 50 or more than 60 years. Serum selenium was positively associated with hemoglobin level in male, age less than 50 years, and participants with high level of dietary iron intake and C‐reactive protein. Furthermore, serum selenium was negatively associated with risk of anemia in participants younger than 50 years old.

**TABLE 3:** Associations between serum selenium and hematological indices of anemia in different subgroups

Subgroup	Serum iron^a^	MCHC^a^	Hemoglobin^a^	Anemia^a^
Age, years				
<50	0.180 (0.032, 0.329) 0.018	0.002 (−0.002, 0.005) 0.374	0.008 (0.003, 0.013) 0.003	0.970 (0.947, 0.994) 0.013
51–60	0.114 (−0.052, 0.280) 0.178	0.002 (−0.002, 0.005) 0.323	−0.001 (−0.007, 0.005) 0.727	1.008 (0.984, 1.032) 0.516
≥60	0.160 (0.088, 0.233) <0.001	0.001 (−0.001, 0.003) 0.202	0.003 (−0.001, 0.006) 0.102	0.994 (0.985, 1.002) 0.146
Gender				
Male	0.131 (0.037, 0.224) 0.006	0.002 (−0.000, 0.004) 0.060	0.004 (0.000, 0.008) 0.029	0.995 (0.984, 1.006) 0.368
Female	0.202 (0.122, 0.282) <0.001	0.001 (−0.001, 0.003) 0.3242	0.003 (−0.000, 0.006) 0.064	0.989 (0.979, 1.000) 0.051
Dietary iron, mg				
Low	0.162 (0.082, 0.243) <0.001	0.001 (−0.001, 0.003) 0.180	0.002 (−0.001, 0.006) 0.160	0.994 (0.984, 1.004) 0.221
High	0.178 (0.083, 0.272) <0.001	0.002 (−0.000, 0.004) 0.128	0.006 (0.002, 0.009) 0.003	0.989 (0.977, 1.001) 0.061
C‐reactive protein, mg/dl				
Low	0.019 (−0.071, 0.108) 0.679	0.003 (0.001, 0.005) 0.007	−0.001 (−0.004, 0.003) 0.768	0.997 (0.986, 1.008) 0.595
High	0.257 (0.174, 0.339) <0.001	0.000 (−0.002, 0.002) 0.953	0.007 (0.003, 0.010) <0.001	0.990 (0.980, 1.000) 0.052

Abbreviations: MCHC, mean corpuscular hemoglobin concentration.

^a^ Adjusted for age, gender, education (less than high school, high school, more than high school, or missing), race (non‐Hispanic white, black, Mexican American, other Hispanic, other race/ethnicity or missing), body mass index (<18.5, 18.5–24.9, 25–29.9, >30, or missing), energy intake, dietary fiber, and dietary iron.

**FIGURE 1:**
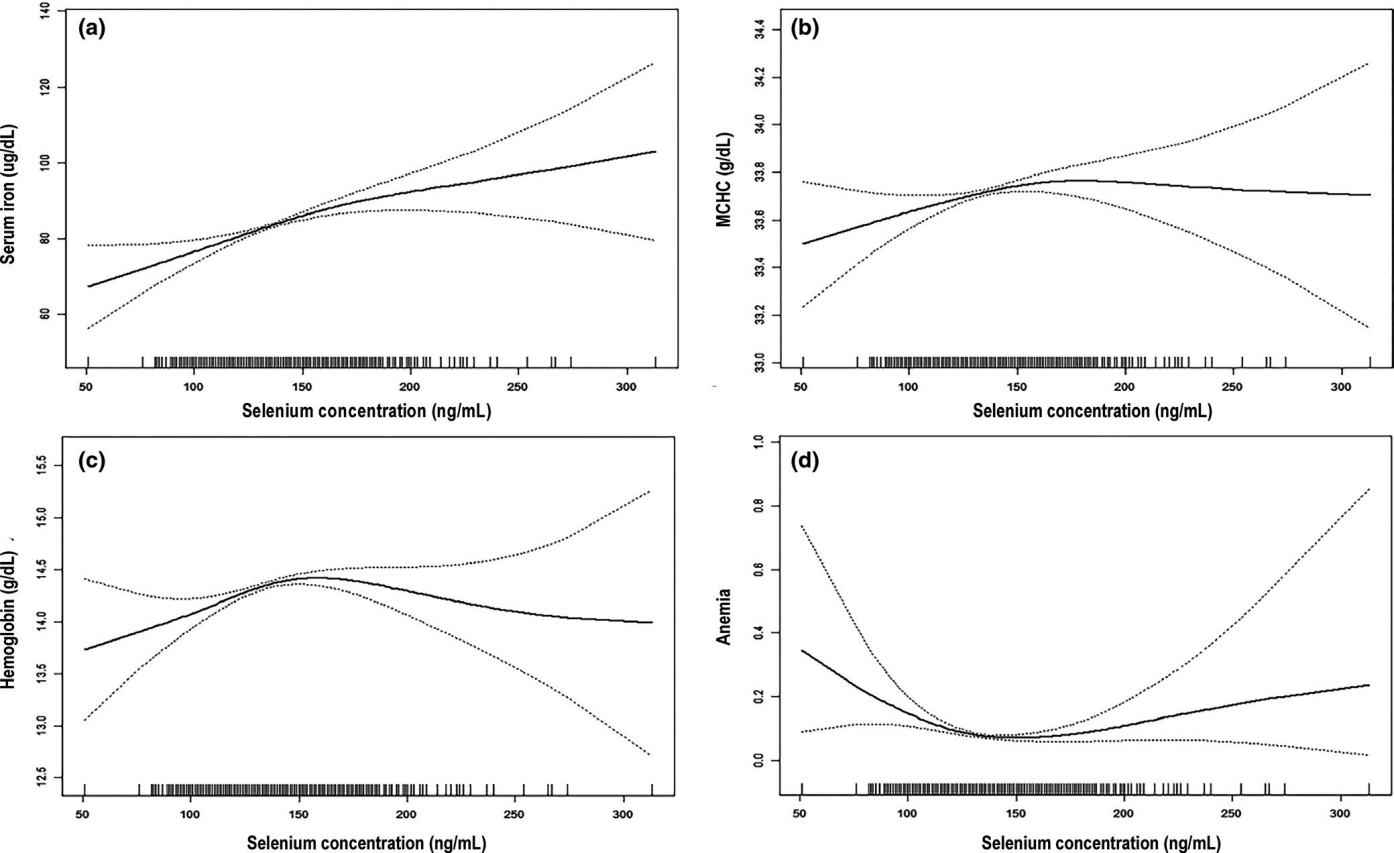
Nonlinear relationship between serum selenium and hematological indices of anemia. (a) Serum iron; (b) MCHC; (c) hemoglobin; (d) anemia. The black solid line represents the fitted line, and the black dotted line represents the 95% CI of the fitted line. Adjusted for age, gender, education (less than high school, high school, more than high school, or missing), race (non‐Hispanic white, black, Mexican American, other Hispanic, other race/ethnicity, or missing), body mass index (<18.5, 18.5–24.9, 25–29.9, >30, or missing), energy intake, dietary fiber, and dietary iron

Adjusted smoothed plots suggested the nonlinear relationships between serum selenium and MCHC, hemoglobin level, and risk of anemia (Figure 1). According to the piecewise linear regression analysis (Table 4), we calculated the turning points were 164 ng/ml in MCHC, 143 ng/ml in hemoglobin, and 138 ng/ml in risk of anemia, respectively. On the left of inflection point (serum selenium concentration < 143 ng/ml), serum selenium was positively associated with hemoglobin level (*β* = 0.010, 95% CI: 0.005, 0.014, *p* < .001). However, on the right of inflection point (serum selenium concentration ≥ 143 ng/ml), the association between serum selenium and hemoglobin concentration was not significant (*β* = −0.003, 95% CI: −0.008, 0.001, *p* = .111). Interestingly, the risk of anemia decreased with increased serum selenium up to the turning point (138 ng/ml) (OR = 0.972, 95%CI: 0.960, 0.985, *p* < .001), and then, the risk of anemia increased with increasing serum selenium concentration (OR = 1.011, 95%CI: 1.002, 1.021, *p* = .023). A linear relationship between the serum selenium and serum iron was observed after adjusting for possible confounders, and log likelihood ratio test result comparing linear model to piecewise model was not significant (*p* = .051).

**TABLE 4:** Threshold effect analysis of serum selenium on hematological indices of anemia with the use of segmented linear regression model

	β/OR (95%CI)^b^	*p‐*value
Serum iron		
<167 ng/ml	0.209 (0.133, 0.285)	<.001
≥167 ng/ml	−0.003 (−0.182, 0.177)	.976
Log likelihood ratio test ^a^	0.051	
MCHC		
< 164 ng/ml	0.003 (0.001, 0.004)	.004
≥164 ng/ml	−0.002 (−0.006, 0.001)	.205
Log likelihood ratio test	0.031	
Hemoglobin		
<143 ng/ml	0.010 (0.005, 0.014)	<.001
≥143 ng/ml	−0.003 (−0.008, 0.001)	.111
Log likelihood ratio test	<0.001	
Anemia		
<138 ng/ml	0.972 (0.960, 0.985)	<.001
≥138 ng/ml	1.011 (1.002, 1.021)	.023
Log likelihood ratio test	<0.001	

^a^ Log likelihood ratio test results comparing linear regression model with two piecewise linear regression model.

^b^ Adjusted for age, gender, education (less than high school, high school, more than high school, or missing), race (non‐Hispanic white, black, Mexican American, other Hispanic, other race/ethnicity, or missing), body mass index (<18.5, 18.5–24.9, 25–29.9, >30, or missing), energy intake, dietary fiber, and dietary iron.

## 4 DISCUSSION

Our cross‐sectional study demonstrated that relative to the lowest quintile of selenium after adjustment for multiple confounding variables, serum selenium concentration is negatively associated with anemia risk and positively associated with serum iron level, mean corpuscular hemoglobin concentration (MCHC), and hemoglobin level. In addition, in reporting on the results of our GAM and piecewise linear regression model, our study is the first to provide comprehensive evidence of the nonlinear relationships of serum selenium level with hemoglobin level, MCHC, and risk of anemia. We determined the locations of inflection points using piecewise linear regression. When the serum selenium concentration was less and more than 143 ng/ml, the hemoglobin level increased and nonsignificantly varied, respectively, with serum selenium concentration. Notably, we also found the risk of anemia decreased with increased serum selenium concentration up to the turning point (138 ng/ml), and then, the risk of anemia increased with the increasing serum selenium when serum selenium concentration was more than 138 ng/ml.

Selenium is part of the selenium‐dependent GPX family (Li et al., 2020), which converts harmful hydrogen peroxide, lipids, and phospholipid hydrogen peroxide into harmless substances (Wang et al., 2017). In the 1990s, some researchers discovered that selenium could regulate the homeostasis of glucose, which is closely related to diabetes, and that the main mechanism is selenium's exertion of an insulin‐like effect, which enhances the activity of insulin receptor kinase and stimulates glucose transport (Wang et al., 2017). A previous meta‐analysis of 16 studies revealed first that a nonlinear relationship exists between serum selenium level and the risk of coronary heart disease (CHD), and second that with serum selenium concentrations of 55–145 µg/L, selenium supplementation reduces the risk of CHD (Zhang et al., 2016). Furthermore, a recent cross‐sectional study reported a negative association between serum selenium level and stroke risk (Hu et al., 2019). Taken together, the aforementioned findings indicate that selenium plays a protective role against many diseases, such as hypertension, diabetes, dyslipidemia, and CHD.

Our study showed that relative to the lowest quintile of selenium, higher serum selenium concentrations are associated with an increased serum iron level, MCHC, and hemoglobin level and a lower risk of anemia. Consistent with our findings, several studies have demonstrated that low serum selenium level is associated with an increased risk of anemia (Nhien et al., 2008; Roy et al., 2012; Semba et al., 2006, 2009). Although the mechanisms underlying this association remain unclear, the antioxidant activity of selenium may play a role in these mechanisms (Roy et al., 2012). The regulation of oxidative stress is an essential requirement for oxygen‐carrying erythrocytes, and a deficiency of antioxidant enzymes reduces the maturity and lifespan of red blood cells (Friedman et al., 2001; Marinkovic et al., 2007). A lower selenium level can upregulate the heme oxygenase‐1 enzyme, which facilitates heme catabolism and subsequently results in the depletion of heme (Mostert et al., 2003). Research has indicated that dietary selenium protects red blood cells from oxidative damage and that a lack of selenoprotein can lead to the hemolysis of red blood cells due to oxidative stress. In addition, selenium deficiency or selenoprotein deficiency seriously impairs stress erythropoiesis and aggravates anemia in rodent models and human patients (Liao et al., 2018). A previous animal experiment suggested that dietary selenium supplementation can improve methimazole‐induced deficiencies in hemoglobin concentration, hematocrit, and number of red blood cells in rats (Amara et al., 2011).

Consistent with the findings of previous studies on the relationships of selenium level with lipid profiles and risks of diabetes and mortality (Bleys et al., 2008; Huang et al., 2020; Lin & Shen, 2021), our study discovered that selenium level had nonlinear relationships with anemia‐related indicators and risk of anemia. Different lines of evidence have suggested that the selenoprotein‐mediated effect is dose‐dependent (Bleys et al., 2008). Studies have reported that the concentration and activity of selenoprotein are maximized when the selenium level is between 70 and 90 ng/ml, and that when the serum selenium level is high, an increase in serum selenium level may not reflect an increase in selenoprotein level or activity (Bleys et al., 2007; Burk, 2002; Lin & Shen, 2021). A recent study suggested that the relationships in selenium nutrition that implicate human health are complex and that the population is vulnerable to geographical deficiency and toxicity due to the narrow physiological range of selenium (Xie et al., 2020), which might explain why high serum selenium exposure is associated with increased anemia risk. Our study provided the first evidence on the nonlinear between serum selenium and hemoglobin level, MCHC, and risk of anemia. However, we also found a linear relationship between the serum selenium and serum iron. We found that the serum selenium was associated with increased serum iron level when the serum selenium concentration was less than 167 ng/ml. In contrast, the serum iron level decreased with increased serum selenium up to the turning point (167 ng/ml) although this association did not reach a statistically significant. The possible explanations might be attributed to the over‐fitting of GAM model due to the limited sample size among population with high serum selenium concentration (≥167 ng/ml).

In our study, we used a GAM and piecewise linear regression model to analyze the nonlinear and linear relationships, respectively, of serum selenium level with anemia‐related hematological indicators and risk of anemia. Because the GAM model performs well in identifying nonlinear relationships, it provides a good approximation of the true relationship between exposure and the corresponding results. However, our study has several limitations. First, our cross‐sectional research design only allowed us to observe the health status of the target population at a single time point and precluded us from making causal inferences. Second, our findings might also have been affected by residual confounding, such as area, economic status, and use of medications. Third, our data set (NHANES data for 2003 to 2004) was small due to limitations of our original data. Although our results can aid scholars and policymakers in public health, they require further validation in future multicenter, prospective, and large‐scale studies.

## 5 CONCLUSION

In conclusion, the results demonstrate that a higher serum selenium concentration is associated with an increased serum iron level, MCHC, and hemoglobin level and a decreased risk of anemia. In addition, our study found the evidence of the nonlinearity of the aforementioned relationships, which would provide new insights on selenium nutrition that implicate anemia and human health. More multicenter, prospective, and large‐scale studies are required to validate our findings.

## CONFLICT OF INTEREST

The authors declare that they do not have any conflict of interest Data availability statement.

## AUTHOR CONTIBUTIONS

QZ and BZZ designed and conceived the study. QYC performed the data analysis. XC and LH revised the manuscript.

## DATA AVALIBLITY STATEMENT

The data that support the findings of this study are available in the DRYAD repository [Patel, Chirag J. et al. (2016), Data from: A database of human exposomes and phenomes from the US National Health and Nutrition Examination Survey, Dryad, Dataset, https://doi.org/10.5061/dryad.d5h62].
